# Global prevalence of temporomandibular disorders: a systematic review and meta-analysis

**DOI:** 10.22514/jofph.2025.025

**Published:** 2025-06-12

**Authors:** Ahmed Yaseen Alqutaibi, Maged S. Alhammadi, Hatem Hazzaa Hamadallah, Ammar Abdulrahman Altarjami, Omar Talal Malosh, Aseel Mohammed Aloufi, Lama Mohammed Alkahtani, Faten Safran Alharbi, Esam Halboub, Abeer A. Almashraqi

**Affiliations:** ^1^Prosthodontics, Substitutive Dental Science Department, College of Dentistry, Taibah University, 41311 Al-Madinah, Saudi Arabia; ^2^Department of Prosthodontics, Faculty of Dentistry, Ibb University, 70270 Ibb, Yemen; ^3^Orthodontics and Dentofacial Orthopedics, Department of Preventive Dental Sciences, College of Dentistry, Jazan University, 82721 Jazan, Saudi Arabia; ^4^College of Dentistry, Taibah University, 41311 Al-Madinah, Saudi Arabia; ^5^Department of Maxillofacial Surgery and Diagnostic Sciences, College of Dentistry, Jazan University, 82721 Jazan, Saudi Arabia; ^6^Department of Clinical Oral Health Sciences, College of Dental Medicine, QU Health, Qatar University, 2713 Doha, Qatar

**Keywords:** Prevalence, Temporomandibular disorders, Female, Age, Myalgia, Arthralgia, South America, Meta-analysis

## Abstract

**Background**: Understanding the global prevalence and characteristics of a given health problem is essential for sizing its global and regional burden, estimating treatment needs, prioritizing healthcare services, and formulating targeted policies. This systematic review and meta-analysis aimed to estimate the global prevalence of temporomandibular disorders (TMDs) by gender, age, and continent, and the prevalence of specific categories such as myalgia, arthralgia, clicking/joint sounds, and limited mouth opening. **Methods**: A comprehensive search was conducted across three databases—PubMed, Scopus and Web of Science and supplemented by manual search up to June 2024. TMD diagnoses were based on the Research Diagnostic Criteria for Temporomandibular Disorders (RDC/TMD) or Diagnostic Criteria for Temporomandibular Disorders (DC/TMD). **Results**: The database search yielded 15,628 records, from which 27 studies involving 20,971 subjects, including 6075 diagnosed with TMDs, were selected for final analysis. All meta-analyses utilized a random effects model. It is estimated that nearly a third of the global population (29.5%) suffers from TMDs. TMDs affected females at a significantly higher rate compared to males (36.7% versus 26.7%), representing a 1.75-fold greater likelihood among females. Prevalence among individuals under 18 years of age is 38.5%, compared to 34.1% in those 18 and older. TMDs are most prevalent in Europe (33.8%), followed by Asia (27.9%) and South America (27.3%); the lowest prevalence was in North America (19.4%). The most frequently reported signs and symptoms of TMDs are myalgia (37.2%), clicking/joint sounds (29.8%), and arthralgia (16.8%), with limited mouth opening/locking being the least prevalent (8.1%). **Conclusions**: TMDs represent a significant and largely unrecognized health burden. Although conducting further primary studies is urgent for confirmation, this current research underscores that TMDs might constitute a silent epidemic that has not garnered the urgent attention it deserves from healthcare providers, the local community, and researchers. **The PROSPERO Registration**: PROSPERO number is CRD42024583777.

## 1. Introduction

The temporomandibular joint (TMJ) is a unique bi-synovial joint with specialized structural elements with highly coordinated and associated movements [1]. The term “temporomandibular disorders” (TMDs) refers to a group of conditions that cause pain and dysfunction in TMJ and the muscles associated with its movement [2]. There are three main classes of TMDs: (1) disorders of the joints, including disc disorders; (2) disorders of the muscles used for chewing (masticatory muscles); and (3) headaches associated with a TMD. Within each class, there are several disorders. TMDs often manifest as pain in the face, jaw, temple and ear regions. The pain may extend to surrounding areas and frequently presents challenges in mouth opening and jaw mobility [2, 3]. Additionally, TMDs are associated with discomfort and difficulty in performing daily activities such as eating, speaking and yawning [4]. Moreover, TMDs are often associated with other health conditions such as headaches, migraines, and sleep disorders. According to the above-mentioned signs, symptoms, and associated comorbidities, TMDs inevitably have a significant impact on an individual’s quality of life [5].

The etiology of TMDs is multifaceted, involving a combination of biological, psychological and socio-environmental factors. Despite extensive research on the topic, the exact causes of TMDs are yet to be completely elucidated [6, 7, 8]. All in all, the etiological factors of TMDs can be classified into three distinct categories: predisposing, initiating and perpetuating factors. While occlusion has been a contentious factor in TMDs, it is not considered the primary etiological factor [9]. Instead, its role may be limited to predisposing individuals to the disorder or perpetuating it. The initiating factors include macro- or micro-trauma and abnormal loading of the masticatory system, whereas the perpetuating factors encompass oral behaviors, emotional disturbances and social influences [10]. The predisposing factors encompass psychological and pathophysiological processes that create a favorable environment for TMD development, potentially leading to chronic pain, the hallmark of TMDs [11].

Understanding the prevalence and characteristics of a given health problem in different populations is essential for sizing its global and regional burden, estimating treatment needs, prioritizing healthcare services, and formulating targeted policies [12]. The same is true for TMDs, which represent a common health problem. Two recent systematic reviews estimated a high global prevalence rate of TMDs, ranging from 31% [13] to 34% [14]. However, the medical literature is full of inconsistencies regarding the global, regional and local burden of TMDs. As a rule of thumb, diagnosing TMDs represents a considerable challenge due to the complexity and variability of their signs and symptoms, the multiple and interrelated nature of etiopathogenesis, and the lack of consistent, valid and reliable criteria for diagnosis. In an attempt to recognize the need for standardized diagnostic criteria, the Research Diagnostic Criteria for Temporomandibular Disorders (RDC/TMD) was developed in 1992, providing a dual-axis approach, with Axis I addressing biological components and Axis II focusing on psychosocial aspects [15]. Despite its initial acceptance, concerns about the sensitivity and specificity of the RDC/TMD led to the development of the Diagnostic Criteria for Temporomandibular Disorders (DC/TMD) [16]. The revised criteria aimed to enhance Axis I’s diagnostic algorithms and incorporate new tools for evaluating Axis II factors. Currently, the DC/TMD is widely regarded as the benchmark for assessing TMDs [16]. The DC/TMD comprises of two main components. Axis I primarily addresses the physical and biological aspects of TMDs, utilizing a symptom questionnaire and a clinical examination protocol to evaluate pain characteristics, range of motion, joint sounds and other signs and symptoms. Accordingly, specific TMD diagnoses are established through the defined diagnostic algorithms based on Axis I assessment. Axis II examines psychological and psychosocial factors associated with TMDs, using standardized questionnaires to assess pain intensity, disability, limitations of jaw functionality, depression, anxiety and parafunctional oral behaviors [16].

Given the inconsistencies reported in the medical literature regarding the application of the two established diagnostic criteria, assessing the prevalence of TMDs among general populations is bound to yield heterogeneous results. Therefore, this systematic review and meta-analysis sought to accurately estimate the global prevalence of TMDs, detailing prevalence rates by gender, age, continent, and specific categories such as myalgia, arthralgia, clicking/joint sounds, and limited mouth opening.

## 2. Methodology

### 2.1 Review protocol and registration

This systematic review adhered to the guidelines outlined in the Preferred Reporting Items for Systematic Reviews and Meta-Analyses (PRISMA) (**Supplementary material 1**) [17]. The review protocol was registered in the International Prospective Register of Systematic Reviews (PROSPERO), with a registration number: CRD42024583777.

### 2.2 Research question and eligibility criteria

The study adopted the following research questions: What is the global prevalence of TMDs? And what are the TMDs prevalence rates by gender, age, continent, and category, following the DC/TMD or RDC/TMD criteria? The review included observational population-based studies, specifically cross-sectional and longitudinal studies that reported the prevalence of TMDs (as the main outcome), with no language or time restrictions. Accordingly, studies were excluded if they were not population-based, did not utilize RDC/TMD or DC/TMD criteria, relied solely on self-reported questionnaires without clinical examinations, did not provide prevalence data, did not focus on TMDs, or were of different design like reviews, case reports, letters or editorial articles.

### 2.3 Information sources and search strategy

Article search was conducted in three electronic databases: PubMed, Scopus and Web of Science, up to June 2024. Additionally, a manual search was performed on reference lists of the relevant publications, along with searching in grey literature through ProQuest Dissertations and Theses Global and conference proceedings. Abstracts of all articles meeting the inclusion criteria were reviewed for eligibility. The search keywords included the following terms either individually or in combination: temporomandibular, TMJ, TMD, joint, disorder, disease, occurrence, frequency, prevalence and incidence. **Supplementary Table 1** provides the keywords used or combined with Boolean operators for each of the databases.

### 2.4 Selection process

The study selection included two distinct phases. First, titles and abstracts were screened independently by three authors (AYA, HHH and AA) to identify the relevant records and eliminate irrelevant ones. Second, the full texts of the selected records were read independently by the same three authors to confirm their suitability. In cases of disagreement, a consensus was reached or a fourth reviewer (MA) was consulted for arbitration.

### 2.5 Data extraction

The data were extracted independently by two reviewers (AYA and OA) using a pre-tested data charting form. The form captured various study characteristics, including unique reference number, last name of the first author, year and place of publication, study setting, sampling design and sample size. In addition, participants’ characteristics were recorded, such as the total number of participants, mean age, and definition of cases. Finally, outcome measures pertaining to the prevalence of TMDs were documented.

### 2.6 Risk of bias assessment

The quality of the included studies was assessed using a modified version of the checklist that was recommended in a recently published systematic review as a comprehensive set of items and domains that is broader than any of the individual tools [18]. This modified checklist consists of 16 items that evaluate various aspects of the studies, including population and setting, condition measurement, and statistical analysis. The quality of each study was then categorized as weak (≤12), moderate (13 to 17), or high (≥18), as outlined in **Supplementary Table 2**.

### 2.7 Data synthesis

The pooled TMD prevalence rates, both overall and per the different grouping factors along with 95% confidence intervals (95% CI) were calculated. Similarly, the pooled odds ratios (ORs) along with 95% CI were calculated to assess the potential association of TMDs with gender. Furthermore, the pooled prevalence rates of the types and signs of TMDs out of the overall reported TMDs were calculated, along with 95% CI. Heterogeneity, if present, was tested using a chi-square test and *I*^2^ statistics: The fixed effects model was used for low/moderate heterogeneity (*I*^2^ ≤ 50%), while the random effects model was applied for significant heterogeneity (*I*^2^ > 50%). Furthermore, the prediction interval (PI) was calculated for each calculated event rate and for the OR in all random effects models to better reflect the real heterogeneity than the chi-square test and *I*^2^ statistics [19]. Publication bias was evaluated using the funnel plot along with Egger’s test. Sensitivity analysis was conducted by excluding studies of low quality (*i.e.*, at high risk of bias), as well as those which prompted discussion during the selection process. Due to the low quality (high risk of bias) of Macrì *et al*.’s [20] study, sensitivity analysis was conducted by excluding this study. All analyses were conducted using the Comprehensive Meta-Analysis Software version 4.0 (Biostat, Inc, Englewood, NJ, USA).

## 3. Results

### 3.1 Study selection

The database search yielded a total of 15,628 records, with 5108 from PubMed, 5965 from Scopus, and 4555 from Web of Science. Duplicate records (2658) were removed, and the remaining 12,970 records were screened by titles and abstracts, upon which 12,823 records were irrelevant and thus excluded. The full texts of the remaining 147 records were retrieved and assessed for eligibility. Of those, 122 were not eligible and were thus excluded; those records along with reasons for exclusion are listed in **Supplementary Table 3**. In addition, 14 records were identified through other resources: 2 from websites and 12 from the reference lists of the included studies. Upon examination of the full text of these 14 studies, 2 were eligible and included, while the other 12 were ineligible and thus excluded. Ultimately, 27 studies [20, 21, 22, 23, 24, 25, 26, 27, 28, 29, 30, 31, 32, 33, 34, 35, 36, 37, 38, 39, 40, 41, 42, 43, 44, 45, 46] encompassing the period from 2008 to 2022 were included in both the qualitative and quantitative synthesis, as indicated in Fig. 1.

**Fig. 1.** PRISMA flowchart of the study search strategy.

### 3.2 Study characteristics

The characteristics of the included studies are presented in Table 1 (Ref. [20, 21, 22, 23, 24, 25, 26, 27, 28, 29, 30, 31, 32, 33, 34, 35, 36, 37, 38, 39, 40, 41, 42, 43, 44, 45, 46]).

**Table 1.** Characteristics of the included studies.

Author (yr)	Country	Continent	Ethnicity	Age (yr)	Sample size	Gender distribution (Males:Females)	Diagnostic tool	Community (schoolchildren, university students, military, *etc.*)	Sampling techniques
Macrì *et al*. [20], 2022	Italy	Europe	NR	7–15	214	NR	DC/TMD	Patients referred to orthodontic clinics (schoolchildren/adolescents seeking orthodontic advice)	Convenience sample
Janal *et al*. [21], 2008	USA	North America	African American, Caucasian	18–75	782	only female	RDC/TMD	Community-women in the NY metropolitan area were selected at random	Random sample
Marklund *et al*. [22], 2008	Sweden	Europe	NR	18–30	308	112:196	RDC/TMD	Students who attended the dentistry program at Umeå University, Umeå, Sweden	Random sample
Balke *et al*. [23], 2010	Iran	Western Asia	Persians	18–65	223	52:171	RDC/TMD	Six healthcare centers/bases in Mashhad, the rural area was from the Zoshk health-care base	Random sample
Moyaho-Bernal *et al*. [24], 2010	Mexico	North America	NR	8–12	235	106:129	RDC/TMD	Attended an official elementary school	NR
Wu *et al*. [25], 2010	China/German	Asia/Europe	German and Chinese	13–18	Germany = 561, China = 497, Total (1058)	554:504	RDC/TMD	Germany: different types of schools from the urban area of Halle (Saale). China: three schools in the city of Jinan (northeast China, in the Shandong province).	Random sample
Franco-Micheloni *et al*. [26], 2015	Brazil	South America	NR	12–14	1307	565:742	RDC/TMD	Public schoolchildren	Random sample
Yu *et al*. [27], 2014	China	Asia	NR	23–52	619	males only	RDC/TMD	Shenzhen Airlines pilots	Random sample
Al-Khotani *et al*. [28], 2016	Saudi Arabia	Asia	NR	10–18	456	184:272	RDC/TMD	Jeddah schools	Random sample
Jussila *et al*. [29], 2017	Finland	Europe	NR	46	1871	867:1004	DC/TMD	Northern Finland birth cohort born in 1966 in Oulu and Lapland provinces	NR
Loster *et al*. [30], 2017	Poland	Europe	Caucasian	16.7–19.3	260	68:192	RDC/TMD	High schools in Krakow, Poland	Random sample
Østensjø *et al*. [31], 2016	Norway	Europe	NR	13–19	562	276:286	DC/TMD	Patients from 4 clinics in Rogaland County, Norway	Random sampling
Bertoli *et al*. [32], 2018	Brazil	South America	NR	10–14	934	416:518	RDC/TMD	Public and private schools in the city of Curitiba, Paraná, Brazil	Stratified random sampling
Talaat *et al*. [33], 2018	United Arab Emirates	Asia	Caucasian	18–65	3009	1832:1177	RDC/TMD	Oral Diagnosis and Urgent Care Clinic at the University Dental Hospital Sharjah, United Arab Emirates	Convenience sampling
Alrashdan *et al*. [34], 2018	Jordan	Asia	NR	18–78	368	183:185	DC/TMD	Patients attending King Abdulla University Hospital	Consecutive sampling
de Melo Júnior *et al*. [35], 2019	Brazil	Europe	NR	10–17	1342	420:922	RDC/TMD	Two regional offices (north and south), 165 public schools, city of Recife	cluster sampling
Jivnani *et al*. [36], 2017	India	Asia	NR	18–28	200	NR	DC/TMD	University students	Simple random sampling
Tecco *et al*. [37], 2019	Italy	Europe	NR	11–19	567	246:321	RDC/TMD	Adolescents were recorded from the dataset in a dental university clinic in Italy	NR
Braido *et al*. [38], 2020	Brazil	South America	NR	12–14	690	301:389	RDC/TMD	Adolescents enrolled in public and private schools	Convenience sampling
Rauch *et al*. [39], 2019	German	Europe	NR	10–18	1116	542:574	DC/TMD	Healthy adolescents	NR
Paduano *et al*. [40], 2020	Southern Italy	Europe	NR	14–18	361	178:183	RDC/TMD	Secondary schools in the city of Catanzaro, Italy	Two-stage cluster sampling
Wieckiewicz *et al*. [41], 2019	Poland	Europe	Caucasian	18– 84	213	64:149	DC/TMD	General population from four Polish cities (Wroclaw, Lublin, Katowice, and Lodz)	Random sampling
Barbosa *et al*. [42], 2021	Portugal	Europe	NR	18– 67	1381	339:1042	RDC/TMD	Students from 19 university institutions from Oporto District, Portugal	NR
Srivastava *et al*. [43], 2021	Saudi Arabia	Asia	NR	20–25	246	137:109	DC/TMD	Dental school students	NR
Wu *et al*. [44], 2021	China	Asia	Han Chinese	University students (age not directly reported)	754	354:400	DC/TMD	Medical university students	Simple random sampling
Alketbi *et al*. [45], 2022	United Arab Emirates	Asia	Asian (53.2%), Whites (39.7%), Others (7.1%)	The mean age was 34.19 ± 10.85	252	137:115	DC/TMD	Students and patients at the College of Dental Medicine, University of Sharjah	Convenience sampling
Progiante *et al*. [46], 2015	Brazil	South America	NR	20–65	1643	56:1083	RDC/TMD	The population was the users of the Brazilian public health system	Public health database

*DC: Diagnostic Criteria; RDC: Research Diagnostic Criteria; TMD: temporomandibular disorders; NR: Not Reported; NY: New York.*

Among the 27 included studies, the RDC/TMD was employed as the diagnostic tool in 17 studies [21, 22, 23, 24, 25, 26, 27, 28, 30, 32, 33, 35, 37, 38, 40, 42, 46], while 10 studies employed the DC/TMD [20, 29, 31, 34, 36, 39, 41, 43, 44, 45].

Twelve studies were conducted in European countries [20, 22, 25, 29, 30, 31, 35, 37, 39, 40, 41, 42], 10 in Asia [23, 25, 27, 28, 33, 34, 36, 43, 44, 45], 4 in South America [26, 32, 38, 46], and 2 in North America [21, 24]. It is essential to emphasize that one study [25] investigated two different populations: Chinese and German, and presented data for each population individually. Therefore, this study is categorized within Asia as Wu *et al*. [25], 2010 (a), and within Europe as Wu *et al*. [44], 2010 (b).

The sample sizes across the 27 studies varied significantly, with the smallest study [36] including 200 participants, and the largest encompassing 3009 participants [33]. The majority of studies had sample sizes ranging between 500 and 3000 participants. Regarding gender composition within the sample, the majority of the studies included both male and female participants. However, a notable trend emerged across many of the studies, with a higher proportion of female participants compared to males [23, 30, 35, 42, 46]. A few studies, however, included a single gender: Yu *et al*.’s [27] study, conducted in China in 2015, included male participants only, and Janal *et al*.’s [21] study included exclusively female participants in the USA.

The prevalence rates of TMDs across the included studies demonstrate considerable heterogeneity, spanning from approximately 10.8% in the United Arab Emirates [33] to 90.7% in Germany [39]. One of the most consistent findings across the reviewed studies was the significant gender disparity in the prevalence of TMDs. Females are more frequently affected by TMDs than males, with some studies reporting that females are up to twice as likely to develop the disorder [26, 32, 33, 35]. Other studies that focused on younger populations, particularly adolescents and young adults, also reported a higher prevalence of TMDs among females [22].

Regarding the type of TMD assessed, the most commonly investigated was myalgia (muscle pain), followed by clicking or noise in TMJ, arthralgia (joint pain), and limited jaw opening or jaw locking. The prevalence of myalgia varied widely between studies, ranging from 9% [23] to 97% [41]. Like myalgia, the prevalence of clicking or noise in TMJ, which is often associated with internal derangement of the joint, ranged from 10% [23] to 97% [41]. The prevalence of arthralgia showed considerable variation across studies, ranging from 4% [30] to 78% [45]. The prevalence of limited jaw opening or jaw locking was found to be less frequent than the other TMDs, ranging from 0.6% [32] to 33% [44]. A few studies investigated combinations of TMDs, for instance, Balke *et al*. [23] reported that 72 out of 223 Iranian participants (approximately 32.3%) experienced facial pain, a symptom that could be related to either myalgia or arthralgia.

### 3.3 Quality assessment

Of the 27 studies, 13 (≈48.1%) were classified as having a low risk of bias, with scores ranging from 18 to 21. In contrast, 13 studies (≈48.1%) displayed a moderate risk of bias, with scores ranging from 13 to 17. Finally, only one study (≈3.8%) was classified as having a high risk of bias, scoring 11 (Table 1).

### 3.4 Meta-analysis

All meta-analyses were conducted using the random effects model, as all *I*^2^ values were larger than 50%. Fig. 2 depicts and summarizes the study’s results regarding the overall global prevalence of TMDs, and the prevalence rates by the different grouping factors.

Based on the 27 studies [20, 21, 22, 23, 24, 25, 26, 27, 28, 29, 30, 31, 32, 33, 34, 35, 36, 37, 38, 39, 40, 41, 42, 43, 44, 45, 46] with 20,971 participants, the global prevalence of TMDs was 29.5% (95% CI = 23.4%–36.4%, PI = 6.8%–70.8%, *I*^2^ = 98.87%; Fig. 3). The publication bias was insignificant (*p* = 0.791; **Supplementary Fig. 1**).

**Fig. 2.** A depiction summarizing the main results of the current meta-analysis (The overall global prevalence of TMDs, and the prevalence rates by different grouping factors). TMD: temporomandibular disorders.

**Fig. 3.** Forest plot for meta-analysis of the global prevalence of TMDs. CI: confidence interval.

The global prevalence of TMDs among males based on 20 studies [22, 24, 25, 26, 27, 29, 30, 31, 32, 33, 35, 36, 37, 38, 39, 40, 42, 43, 44] including 7250 male participants was 26.7% (95% CI = 18.2%–37.3%, PI = 3.3%–79.7%, *I*^2^ = 98.37%; Fig. 4). Publication bias was insignificant (*p* = 0.956; **Supplementary Fig. 2**). The global prevalence of TMDs among females based on 20 studies including 8674 female participants [21, 22, 24, 25, 26, 29, 30, 31, 32, 33, 35, 36, 37, 38, 39, 40, 42, 43, 44] was 36.7% (95% CI = 27.9%–46.4%, PI = 7.7%–80.2%, *I*^2^ = 98.4%; Fig. 5). Publication bias was also insignificant (*p* = 0.445; **Supplementary Fig. 3**). Based on 19 studies including 6631 males and 7892 females [22, 24, 25, 26, 27, 29, 30, 31, 32, 33, 35, 36, 37, 38, 39, 40, 42, 43, 44], the prevalence of TMDs among females was significantly 1.75-fold higher than the prevalence among females (OR = 1.75; 95% CI = 1.34–2.33; *p* < 0.001), although the PI of that OR was not significant (0.52–5.92, *I*^2^ = 89.41%; Fig. 6). The publication bias was also insignificant (*p* = 0.936; **Supplementary Fig. 4**).

**Fig. 4.** Forest plot for meta-analysis of the global prevalence of TMDs among males.

**Fig. 5.** Forest plot for meta-analysis of the global prevalence of TMDs among females. CI: confidence interval.

**Fig. 6.** Forest plot for meta-analysis of the comparison of the global prevalence of TMDs by gender. CI: confidence interval.

Sensitivity analysis was conducted by excluding Macrì *et al*. [20] due to its low quality (*i.e.*, high risk of bias). There were no substantial changes in the overall prevalence rate of TMDs or any of its subtypes after excluding the study compared to the results when it was included (**Supplementary Table 4**).

The prevalence of TMDs was found to vary by continent (Fig. 7), with the highest rate in Europe [20, 22, 29, 30, 31, 35, 37, 39, 40, 41, 42] (33.8%; 95% CI = 22%–48%, PI = 4.3%–85%; number of studies (NS) = 11, number of participants (NP) = 8756), followed by Asia [20, 22, 29, 30, 31, 35, 37, 39, 40, 41, 42] (27.9%; 95% CI = 18.7%–39.5%, PI = 4.9%–74.4%; NS = 10, NP = 6624) and South America [26, 32, 38, 46] (27.3%, 95% CI = 21.4%–34%, PI = 7.5%–63.5%, NS = 4, NP = 4574), while the lowest prevalence rate was in North America [21, 24] (19.4%, 95% CI = 5.5%–49.8%, NS = 2, NP = 1017). The prevalence rates of TMDs among males and females in different continents are presented in **Supplementary Figs. 5,6**, respectively.

**Fig. 7.** Forest plot for meta-analysis of the prevalence of TMDs by continent. CI: confidence interval.

The global prevalence of TMDs among those under 18 years of age based on 14 studies [20, 24, 25, 26, 28, 31, 32, 35, 37, 38, 39, 40, 44] including 8091 participants was 38.5% (95% CI = 25.3%–53.7%, PI = 4.8%–88.7%; *I*^2^ = 98.01%; Fig. 8), which is slightly higher than the prevalence among those older than 18 years based on 14 studies [21, 22, 23, 27, 29, 30, 33, 34, 36, 41, 42, 43, 45, 46] including 8691 participants (34.1%), although the 95% CI and PI were narrower (26%–43.4% and 8.9%–73.2%, respectively; *I*^2^ = 98.36; Fig. 9). These studies did not show publication bias (*p* = 0.943 and 0.4696, respectively; **Supplementary Figs. 7,8**, respectively). As none of the studies included reported both age groups, no meta-analysis was conducted in this respect.

**Fig. 8.** Forest plot for meta-analysis of the global prevalence of TMDs among children and adolescent (≤18 years). CI: confidence interval.

**Fig. 9.** Forest plot for meta-analysis of the global prevalence of TMDs among adults (≥18 years). CI: confidence interval.

The prevalence rates of different diagnostic tools are presented in Fig. 10. The prevalence based on DC/TMD (35.4%; 95% CI = 18.8%–56.5%, PI = 1.9%–94%, NS = 10, NP = 5796; *I*^2^ = 99.36%) was higher but less precise than the rate of RDC/TMD (26.5%, 95% CI = 21.3%–32.5%, PI = 8.6%–57.9%, NS = 18, NP = 15175; *I*^2^ = 98.22%).

**Fig. 10.** Forest plot for meta-analysis of the global prevalence of TMDs by the diagnostic tools (DC/TMD versus RDC/TMD). CI: confidence interval.

The prevalence rates of different types and signs of TMDs were as follows: The global prevalence of myalgia based on 19 studies [20, 23, 28, 29, 30, 31, 32, 33, 34, 35, 37, 39, 40, 41, 42, 43, 44, 45, 46, 47] including 6042 participants was 37.2% (95% CI = 27.3%–48.3%, PI = 6.4%–83.6%; *I*^2^ = 97.98%; Fig. 11), with no publication bias (*p* = 0.206; **Supplementary Fig. 9**). The global prevalence of arthralgia based on 18 studies [20, 23, 28, 29, 30, 31, 32, 33, 34, 35, 36, 39, 40, 41, 42, 43, 45, 46] including 5587 participants was 16.8% (95% CI = 9.4%–28.3%, PI = 1%–80.7%; *I*^2^ = 98.33; Fig. 12), with no publication bias (*p* = 0.519; **Supplementary Fig. 10**). The global prevalence of clicking/sound based on 22 studies [20, 23, 25, 27, 28, 29, 30, 31, 32, 33, 34, 35, 36, 37, 39, 40, 41, 43, 44, 45, 46] including 6372 participants was 29.8% (95% CI = 23.9%–36.5%, PI = 9.1%–64.4%; *I*^2^ = 95.72; Fig. 13), also with no publication bias (*p* = 0.942; **Supplementary Fig. 11**). The global prevalence of limited mouth opening based on 19 studies [20, 25, 27, 28, 29, 30, 32, 33, 34, 35, 37, 39, 40, 41, 43, 44, 45, 46] including 6076 participants was 8.1% (95% CI = 5%–12.9%, PI = 0.9%–47.4%; *I*^2^ = 96.48; Fig. 14), although the included studies showed significant publication bias (*p* = 0.003; **Supplementary Fig. 12**).

**Fig. 11.** Forest plot for meta-analysis of the global prevalence of myalgia. CI: confidence interval.

**Fig. 12.** Forest plot for meta-analysis of the global prevalence of arthralgia. CI: confidence interval.

**Fig. 13.** Forest plot for meta-analysis of the global prevalence of clicking/sound. CI: confidence interval.

**Fig. 14.** Forest plot for meta-analysis of the global prevalence of limited mouth opening. CI: confidence interval.

## 4. Discussion

This systematic review and meta-analysis aimed to estimate the worldwide prevalence of TMDs diagnosed using either the RDC/TMD or DC/TMD criteria [15, 16]. This study estimates that nearly a third of the global population (29.5%) suffers from TMDs, with a higher prevalence among females than males, and among individuals under 18 years of age. Regional variations were observed, with the highest prevalence in Europe and the lowest in North America. Myalgia was the most common symptom, while limited mouth opening/locking was the least prevalent.

Although Zieliński *et al*. [14] conducted a recent meta-analysis on the global prevalence of TMDs utilizing 74 records, we think that this current study is more robust in many aspects (refer to the inclusion criteria of the potential studies). In brief, the studies that were included are those with a sample size of at least 200 subjects; based the diagnosis on the Research Diagnostic Criteria for Temporomandibular Disorders (RDC/TMD) or Diagnostic Criteria for Temporomandibular Disorders (DC/TMD); and conducted clinical examination. Further, our study applied a more robust tool to assess the risk of bias. Hence, the included studies are more homogenous, consistent, and reliable. Furthermore, our study included analyses for the different types of TMDs, not included in Zieliński *et al*.’s [14] study.

In fact, using standardized international diagnostic criteria such as the RDC/TMD and DC/TMD, which we set as an inclusion criterion, highlights the importance of reliable assessment methods. The more reliable and valid the diagnostic tool, the more accurate the diagnosis will be. Our study was strict with regard to the tool of TMD diagnosis. This standardization facilitates the comparability of results across different populations and settings, thereby enhancing the generalizability of the findings. The consistent use of these diagnostic criteria aids clinicians in accurately identifying and managing TMDs, ensuring that patients receive appropriate and evidence-based care. From a research perspective, the continued use of standardized diagnostic criteria is crucial for maintaining consistency across studies and improving the quality and accuracy of prevalence estimates for TMDs [16, 33, 48].

It seems that TMDs are not uncommon: Nearly a third of the global population (29.5%) are estimated to have TMDs, with males affected less frequently than females (26.7% versus 36.7%; OR = 0.569; *p* < 0.001). It has been reported in the medical literature that the rates of TMDs are higher among individuals under 18 years of age. Our meta-analysis corroborates this finding, revealing a prevalence of 38.5% among those under 18 years, in contrast to a prevalence of 34.1% among individuals aged 18 years and older. TMDs appear to be predominantly concentrated in Europe (33.8%), followed by Asia (27.9%) and South America (27.3%), whereas their prevalence is comparatively lower in North America (19.4%). As expected, the most prevalent TMD types are myalgia (37.2%), clicking/joint sound (29.8%), and arthralgia (16.8%), while limited mouth opening/locking was the least prevalent (8.1%). The availability of such data is of paramount importance for sizing the burden of TMDs, estimating treatment needs, allocating resources, and informing targeted policies and interventions.

Based on these results, TMDs might represent a significant and overlooked burden. This conclusion is supported by the global burden of oral conditions data from 1990 to 2017 [49], which indicated a global prevalence of dental caries in permanent dentition of 29.4%, periodontitis of 9.8%, and total tooth loss of 3.3%. In contrast, our systematic review reveals a TMD prevalence rate that is similar to or even surpasses these global burdens, highlighting that TMDs might constitute a silent epidemic that has not received the necessary attention from healthcare providers, the local community, or researchers. However, further robust research is urgently needed to confirm or refute such a conclusion.

Our meta-analysis found that the global prevalence of TMDs is 29.5%, based on combined results from both RDC/TMD and DC/TMD tools. However, the prevalence was 35.4% based on DC/TMD and 26.5% based on RDC/TMD. Although the number of participants in the studies (n = 18) that used the old version of the tool (RDC/TMD) was 15175, nearly three times the number of participants in the studies (n = 10) that used the new version (DC/TMD, 5796 participants), this is yet not a logic explanation of the observed difference. Instead, given that DC/TMD is an updated tool of the previous one (RDC/TMD), it is expected to have higher diagnostic accuracy, better sensitivity, and specificity. Our observation implies that the sensitivity (ability to distinguish those who have TMDs as diseased with TMDs) and specificity (ability to distinguish those with no TMDs as healthy) have been substantially improved leading to more true positive and less false positive detection, and we believe this is why the prevalence rate is higher with the new tool (DC/TMD). We quote herein what Schiffman and colleagues, who developed these tools, stated in their publication: “The original RDC/TMD Axis I physical diagnoses have content validity based on the critical review by experts of the published diagnostic approach in use at that time and were tested using population-based epidemiologic data. Subsequently, a multicenter study showed that, for the most common TMD, the original RDC/TMD diagnoses exhibited sufficient reliability for clinical use”. They also mentioned that the new DC/TMD needs to be evaluated for a long time in future studies [16]. Accordingly, the old tool might have led to underestimation. However, the new tool can’t be innocent of overestimation. Generally, we are still waiting for further studies to determine the validity of the new tool exactly as we previously judged the old tool.

In their review, Valesan *et al*. [13] reported an overall prevalence of TMDs in adults and the elderly of 29.3% for RDC/TMD, 38.8% for DC/TMD, and 31.1% for the combined criteria (RDC + DC). In another review, Zieliński *et al*. [14] noted a global TMD incidence of 34%. The former analysis included 21 studies using five different search engines, while the latter comprised 74 studies sourced only from PubMed over a three-year period, highlighting inconsistencies in study selection criteria. In our study, we applied strict selection guidelines, where a minimum of 200 participants was conditional, whereas the previous studies included research with as few as 30 participants, which may not accurately represent the broader population. The high prevalence of TMD cases in the population highlights the significance of this condition. It poses a considerable economic and social challenge. Continued research into its diagnosis and treatment is essential.

Studies consistently show that females are more affected than males, with some research suggesting that females are twice as likely to develop TMDs [29, 50, 51]. Our study supports these findings: males were found to be affected less frequently than females (26.7% versus 36.7%; OR = 0.569; *p* < 0.001). In their review, Zieliński *et al*. [14] concluded that, on average, the female group was 9% to 56% larger than the male group across all continents. These findings highlight the need for targeted interventions focusing on females to help reduce the burden of TMDs in this vulnerable group [50]. The hormonal and psychosocial factors associated with TMDs could offer valuable insights into the observed gender differences, potentially guiding more effective prevention and treatment strategies [52]. The substantial disparities between genders underscore the importance of implementing gender-specific treatment approaches to target the elevated prevalence of TMDs among females, potentially leading to enhanced patient outcomes.

The prevalence of TMDs is reportedly higher in early adulthood, particularly between the ages of 20 and 40, and tends to decrease in older adults. In their review, Valesan *et al*. [13] were unable to conduct a meta-analysis for TMD prevalence in children, while Zieliński *et al*. [14] reported rates of 27% for those under 18 and 41% for those between 18 and 60 years. Our meta-analysis revealed slightly higher prevalence of TMDs among individuals under 18 years of age (38.5%), compared to 34.1% among individuals aged 18 years and older.

TMDs appear to be most prevalent in Europe (33.8%) followed by Asia (27.9%) and South America (27.3%), with the lowest prevalence rate was in North America (19.4%). These findings do not align with certain results reported by Zieliński *et al*. [14], who noted prevalence rates of 33% in Asia, 29% in Europe, 47% in South America and 26% in North America. This discrepancy raises important questions about the relationship between TMDs and geographic, cultural, or anthropometric factors. Additionally, the lack of data from Africa and Australia, along with the limited number of studies in North and South America, highlights the need for greater attention from researchers and healthcare providers in these regions.

The lower reported prevalence of TMD in North America should be interpreted with caution in the context of the findings of the present meta-analysis, as it is based on data from only two studies. This observation does not necessarily suggest a lower true prevalence; rather, it underscores the need for further research to attain a more comprehensive understanding of TMD prevalence in this region.

The most common types of TMDs were myalgia (37.2%), clicking/joint sounds (29.8%), and arthralgia (16.8%), while limited mouth opening/locking was the least common (8.1%). Recent systematic reviews did not report the prevalence of myalgia; Valesan *et al*. [13] only mentioned clicking/joint sounds and arthralgia, reporting the prevalence of the former at approximately 25.9% and of the latter at just 7%. Their analysis included only 11,535 participants, whereas our meta-analysis included 20,971 individuals, making our results more representative of the population due to the larger sample size.

This systematic review and meta-analysis offer a thorough overview of the prevalence of TMDs across various populations and settings. The findings highlight the necessity for targeted interventions and additional research to effectively tackle the global burden of TMDs. Generally, studies with larger sample sizes were found to yield more reliable and accurate prevalence estimates, underscoring the importance of larger samples in refining prevalence data. This is essential for developing effective treatment strategies for TMDs.

## 5. Strengths and limitations

One notable strength of this study compared to previous reviews is its adherence to strict criteria, particularly in terms of diagnostic standards (RDC/TMD and DC/TMD). Other strengths include a minimum sample size of 200 and a focus on population-based studies. However, there are limitations to acknowledge in the present study. Relying on only three databases may have led to the omission of some relevant studies, even though these databases are among the most comprehensive in the field. Other databases like Embase or CINAHL might improve the comprehensiveness of the findings. The presence of one study with a high risk of bias, along with 13 with a moderate risk of bias, is a significant limitation, though it reflects transparency in reporting the various aspects of the current review. Other quality assessment tools like Joanna Briggs Institute (JBI) or the Newcastle-Ottawa Scale (NOS) might reveal different findings. Another limitation worth mentioning was the high heterogeneities of the outcomes among studies which might raise doubts about the results of meta-analyses, and hence weaken the conclusions. Furthermore, this review included studies that involved student populations, particularly within school settings, which may limit the generalizability of our findings due to the typically higher prevalence of TMD among these groups compared to the general population. However, we incorporated studies with a minimum sample size of 200 to strengthen the robustness of our results. Furthermore, the reliance on school-based samples represents a pragmatic approach to investigating younger age cohorts, particularly those under 18 years. Future research should prioritize sampling from broader population cohorts to ensure the findings reflect the general population. Moreover, while our study aimed to illustrate age-specific trends, we recommend that future meta-analyses differentiate between student and general population samples to stand on these prevalence rates and associations across these demographics more effectively. This approach would enhance the applicability of findings to both clinical practice and public health interventions aimed at addressing TMD in diverse age and population groups.

## 6. Conclusions

This comprehensive review and meta-analysis of 27 studies examining the global prevalence of TMDs provides critical insights into the distribution, demographics, and characteristics of TMDs across different populations. However, given the relatively few number of studies included that might not represent the different geographic regions, populations, and types of TMDs, and the other limitations mentioned earlier, there is an urgent need for further research to confirm or refute the below-listed conclusions, and to interpret them cautiously:

1. TMDs affect nearly a third of the global population (29.5%), indicating that one in nearly three individuals may be affected by these disorders worldwide.

2. The global burden of TMDs is higher among females compared to males (36.7% versus 26.7%).

3. TMDs are slightly more prevalent among those under 18 years, with a prevalence of 38.5% among those under 18 years of age compared to 34.1% among those aged 18 years and older.

4. TMDs’ burden varies significantly by continent, with higher prevalence in Europe (33.8%) followed by Asia (27.9%) and South America (27.3%), with a lower prevalence in North America (19.4%).

5. The most common types and symptoms of TMDs are myalgia (37.2%), clicking/joint sounds (29.8%), arthralgia (16.8%), and limited mouth opening (8.1%).

## Supplementary Material

Supplementary material associated with this article can be found, in the online version, at https://files.jofph.com/files/article/1933032104412495872/attachment/Supplementary%20material.zip.






