# Supplementary material for: Global prevalence of temporomandibular disorders: a systematic review and meta-analysis
Source: J Oral Facial Pain Headache. 2025 Jun 12;39(2):48–65. doi: 10.22514/jofph.2025.025 (PMC12531580; doi:10.22514/jofph.2025.025)
Supplement: Supplementary file 1 [file Supplementary-Figs.docx]

Supplementary material

**
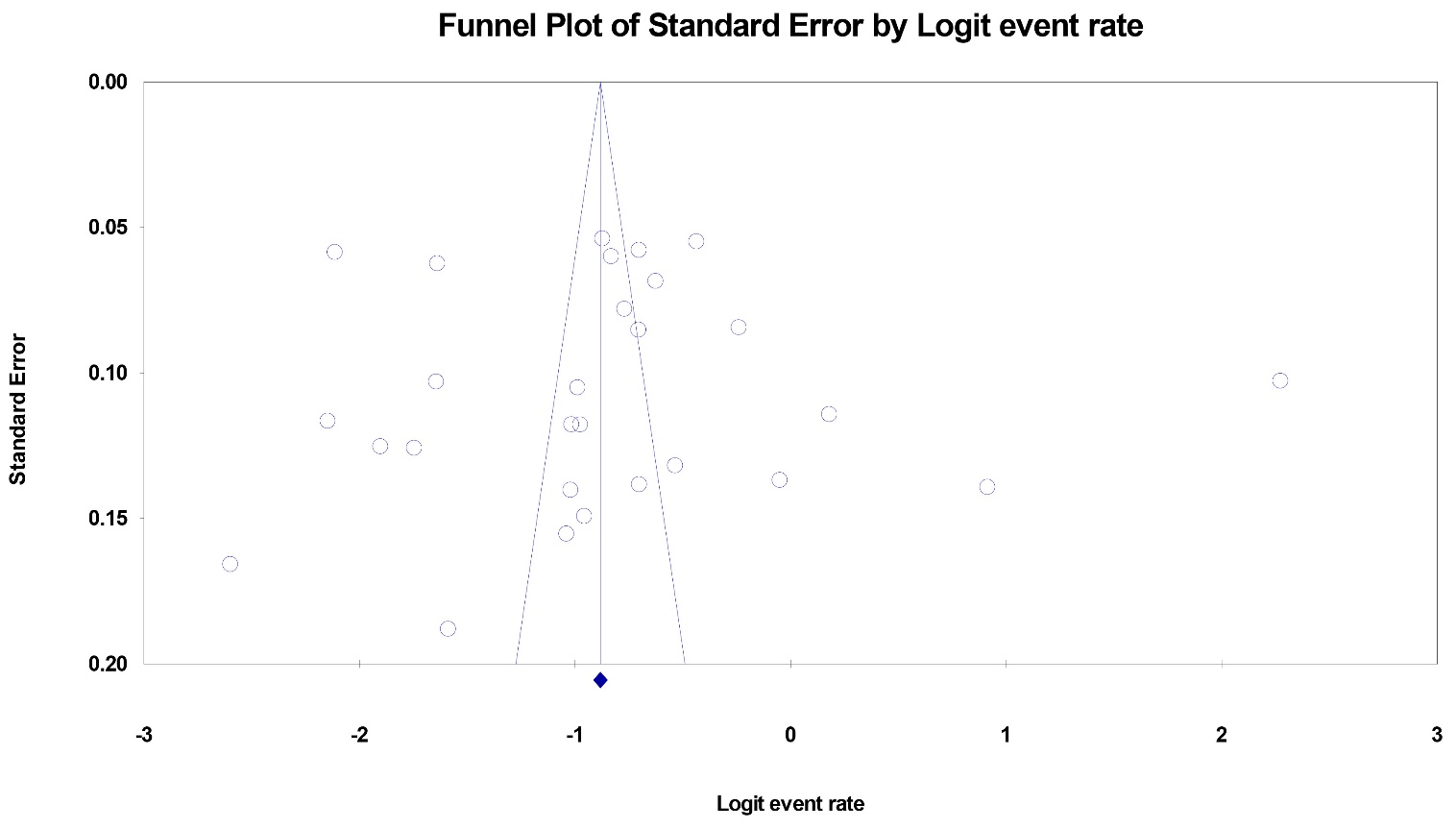
**

Supplementary Fig. 1. Funnel plot for publication bias of the global prevalence of TMDs.

**
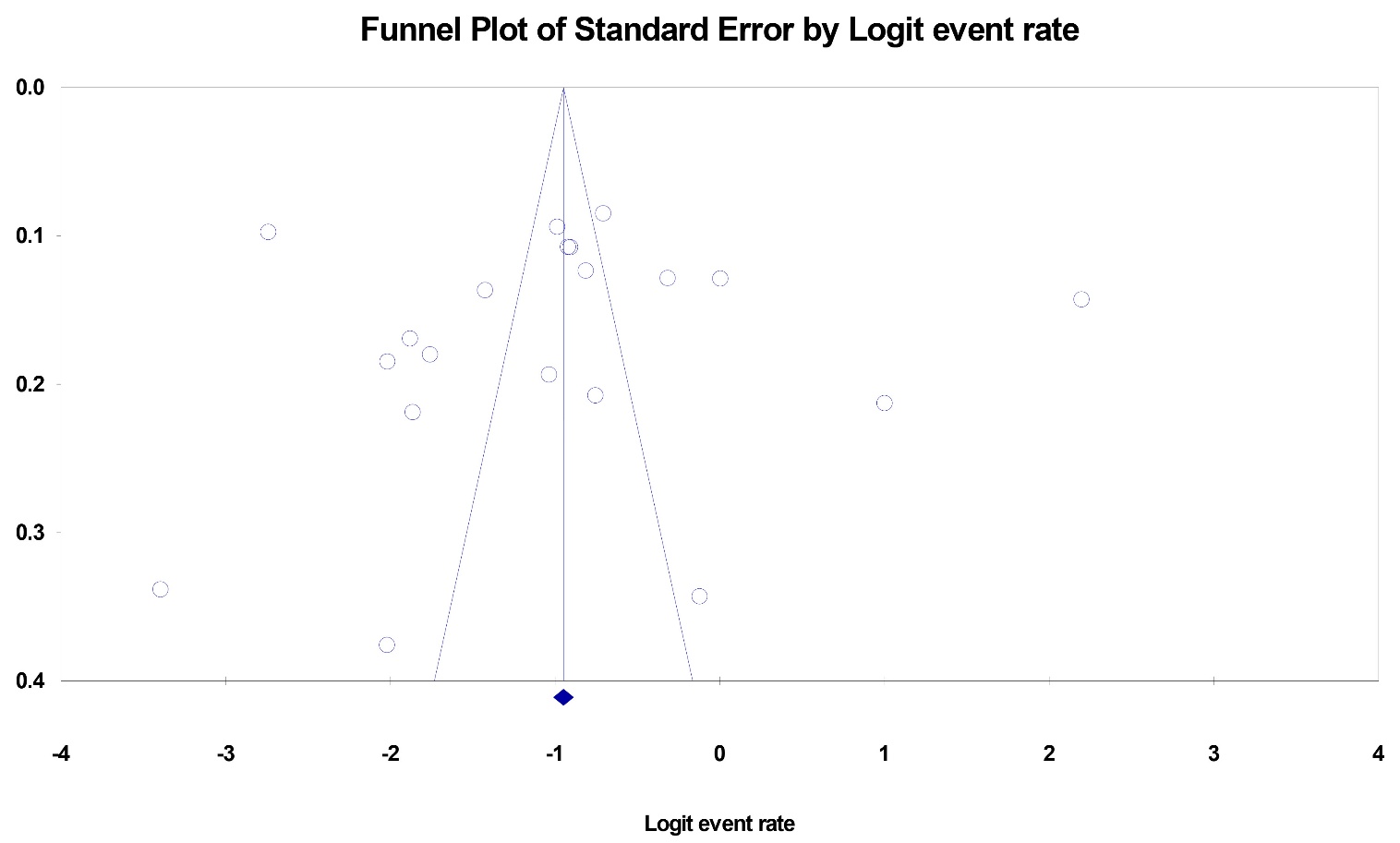
**

Supplementary Fig. 2. Funnel plot for publication bias of the global prevalence of TMDs among males.

**
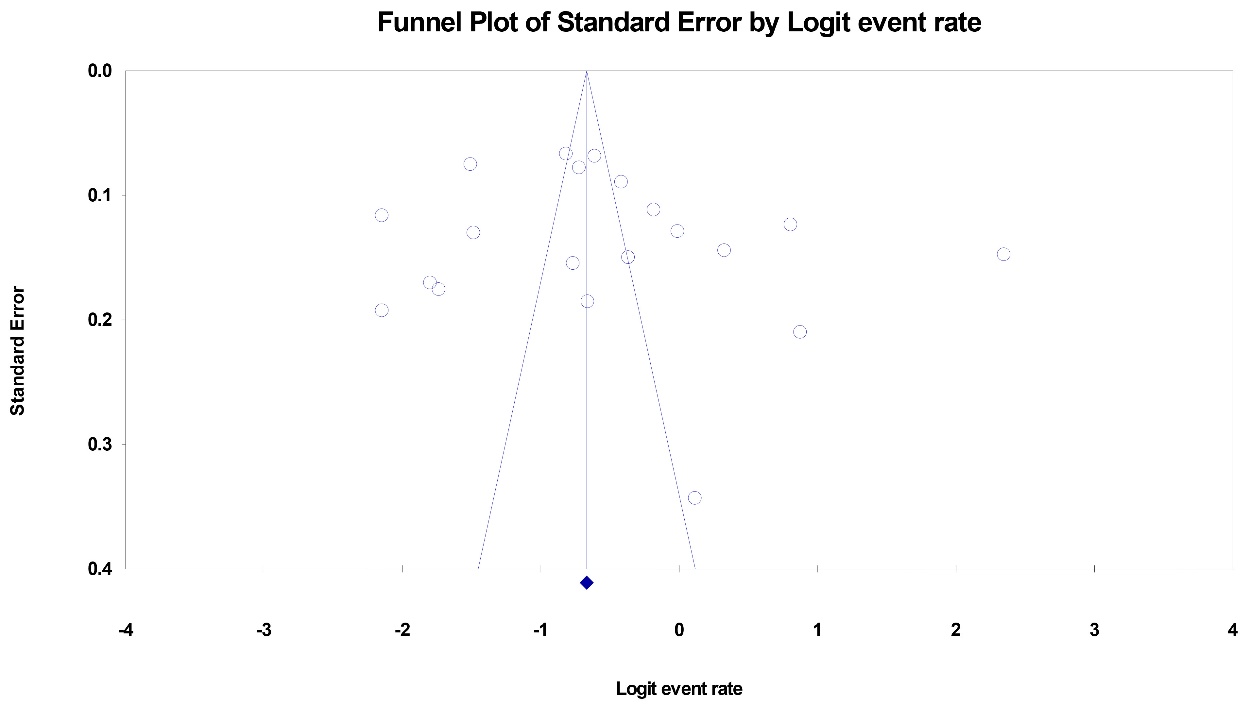
**

Supplementary Fig. 3. Funnel plot for publication bias of the global prevalence of TMDs among females.

**
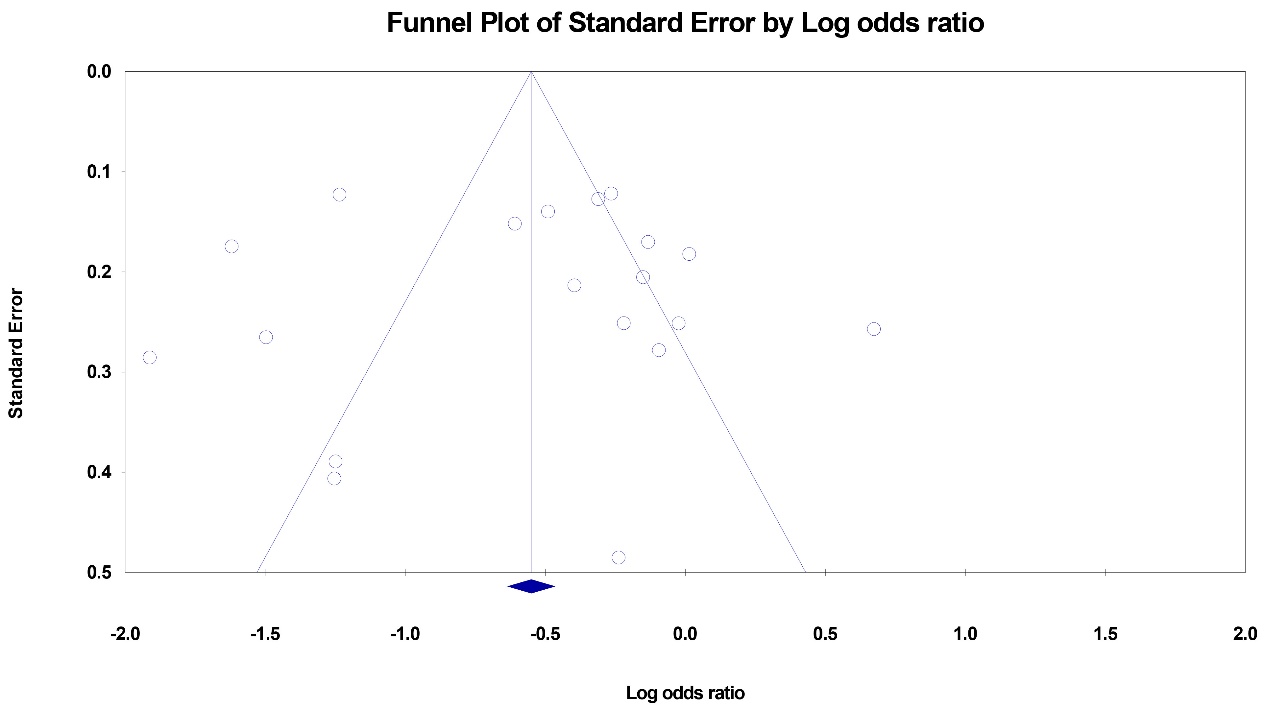
**

Supplementary Fig. 4. Funnel plot for publication bias of the global prevalence of TMDs by continents.

**
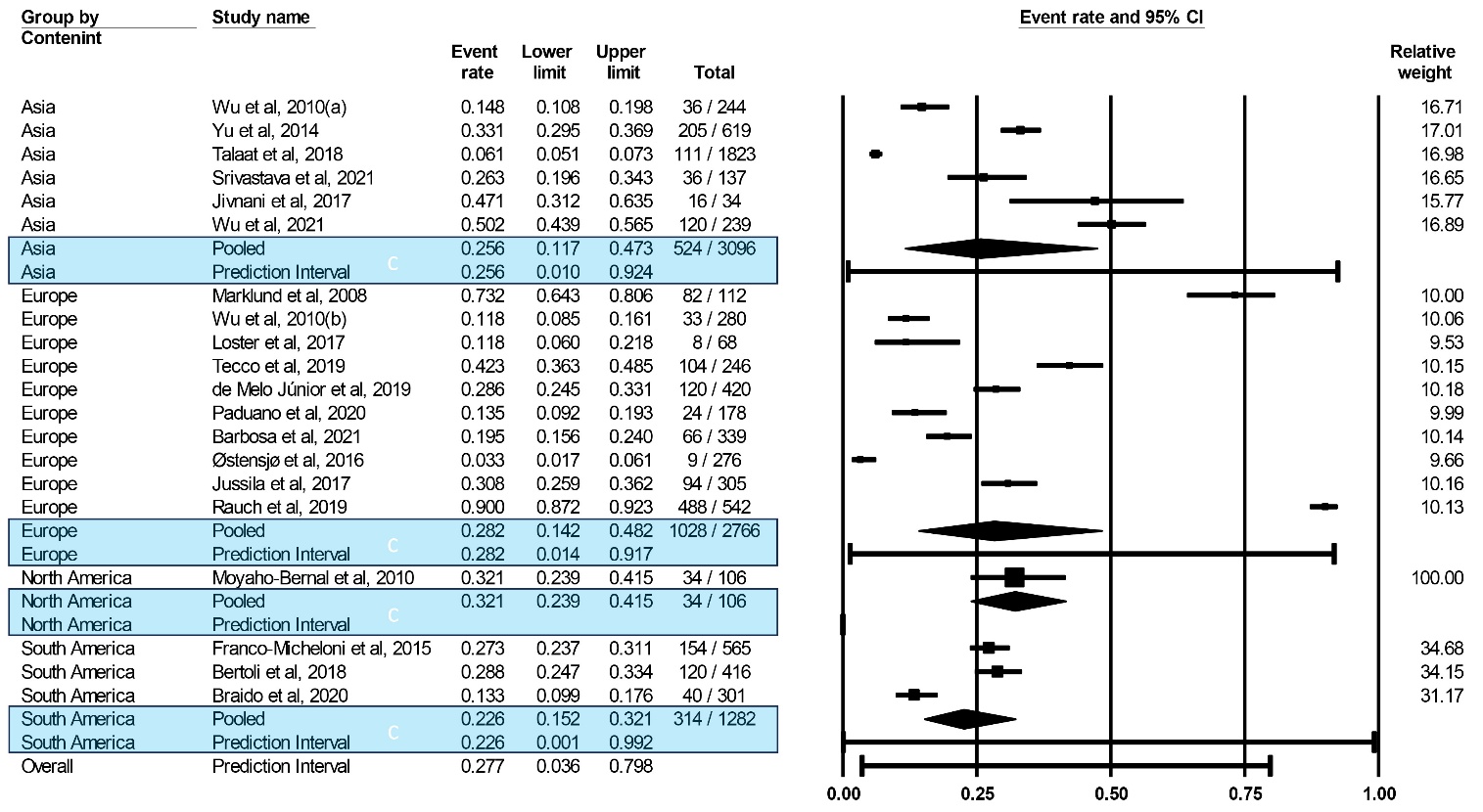
**

Supplementary Fig. 5. Forest plot for meta-analysis of the global prevalence of TMDs among males in different continents.

**
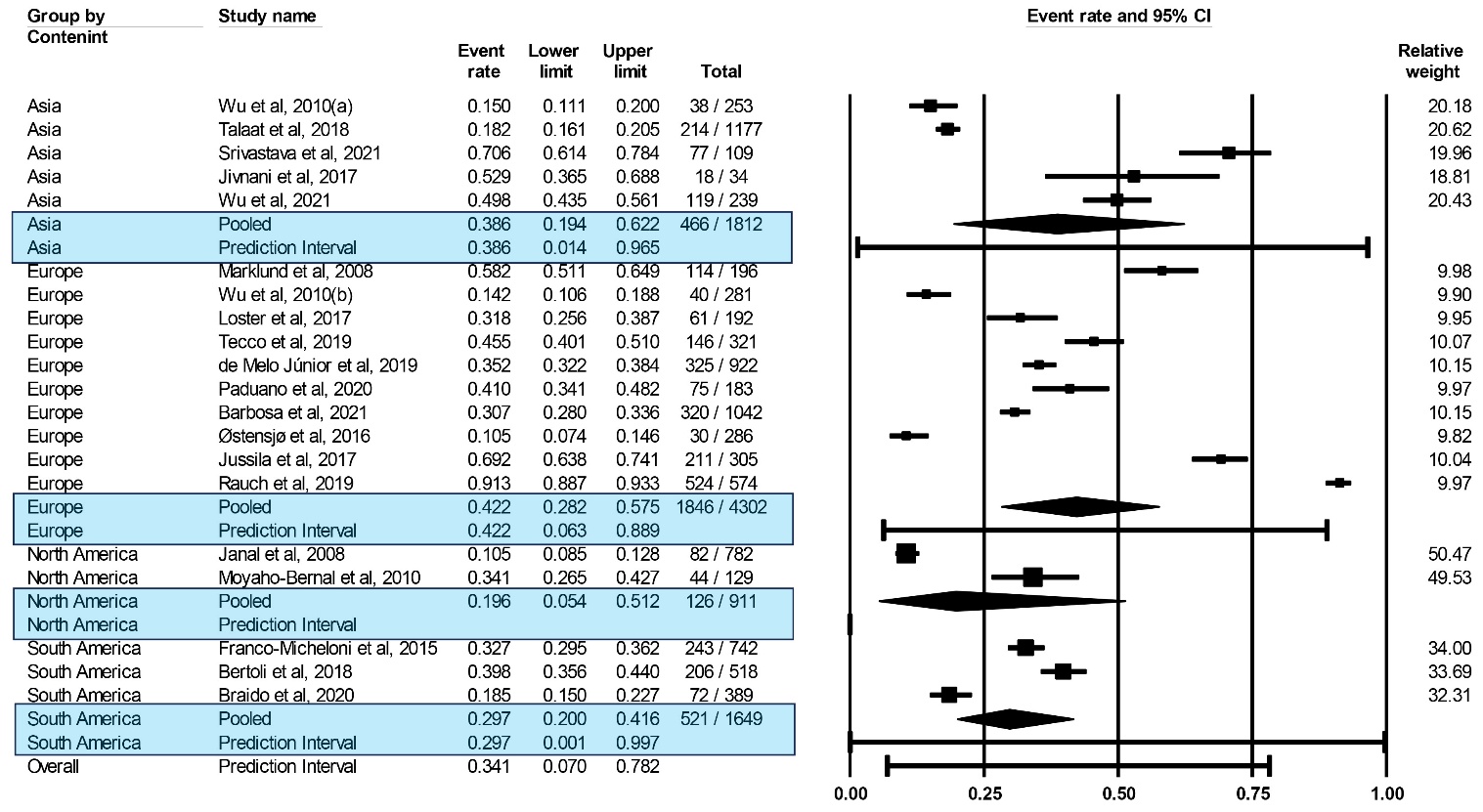
**

Supplementary Fig. 6. Forest plot for meta-analysis of the global prevalence of TMDs among females in different continents.

**
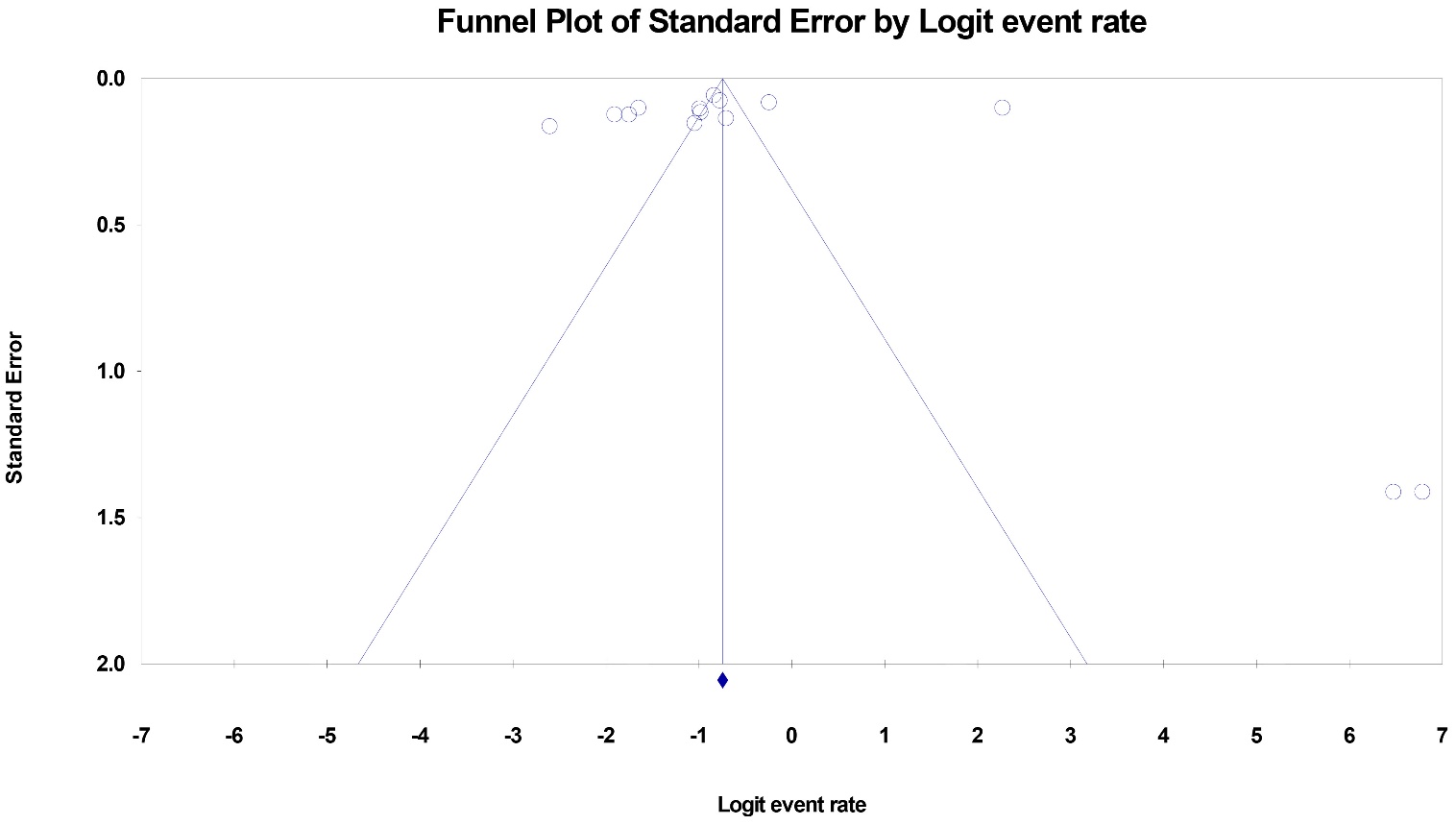
**

Supplementary Fig. 7. Funnel plot for publication bias of the global prevalence of TMDs among 18 years old and younger.

**
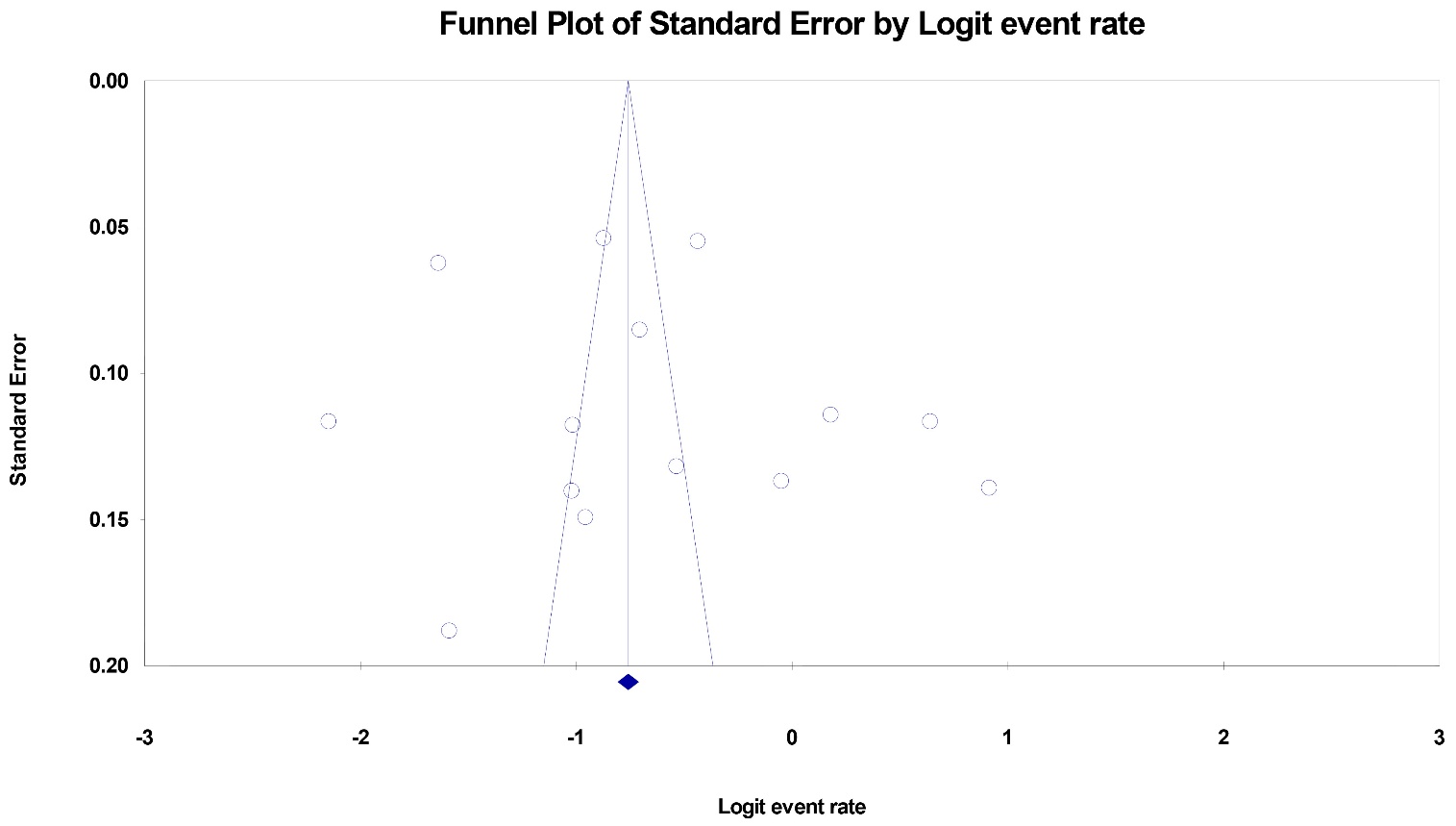
**

Supplementary Fig. 8. Funnel plot for publication bias of the global prevalence of TMDs among older than 18 years old.

**
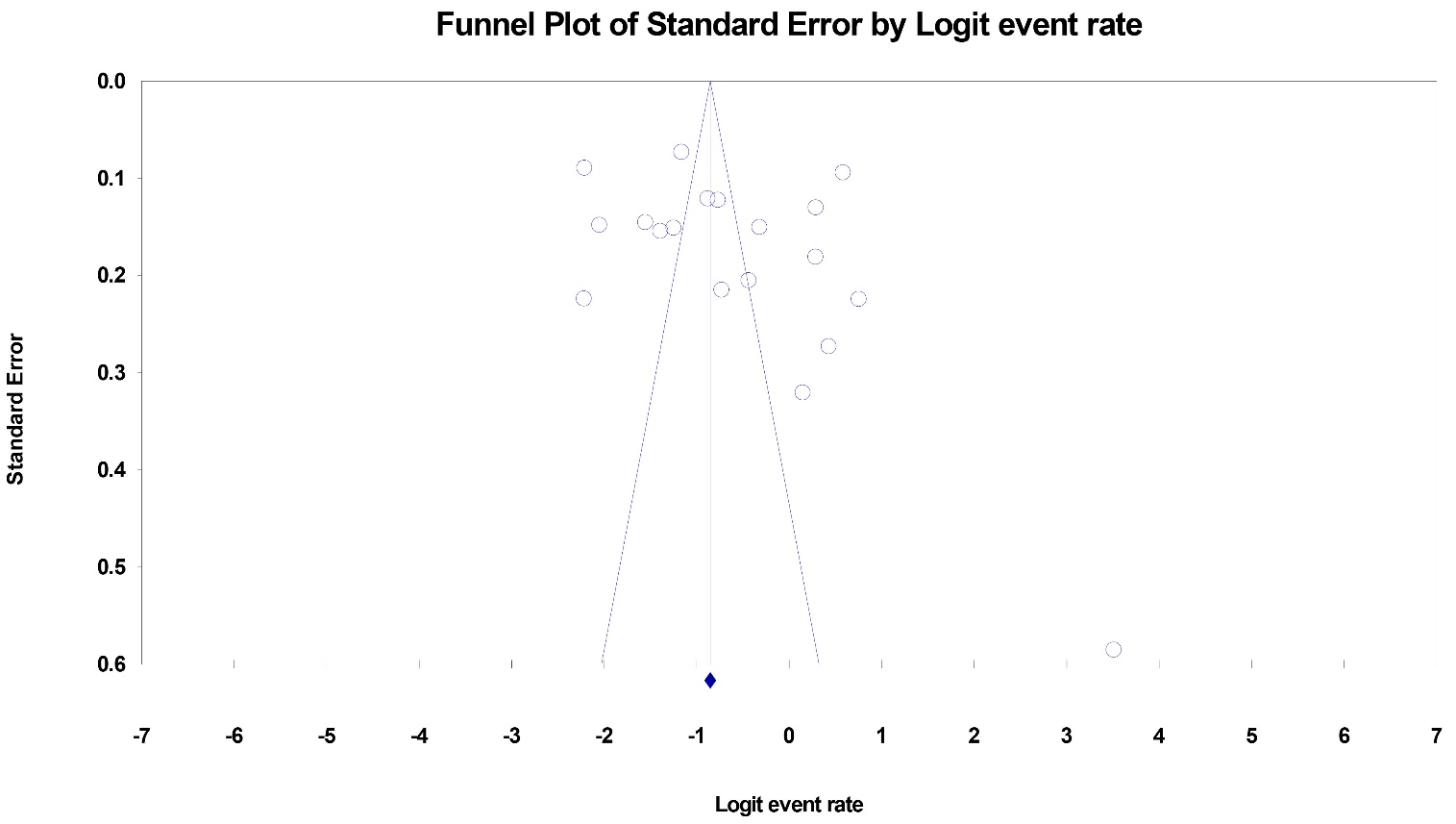
**

Supplementary Fig. 9. Funnel plot for publication bias of the global prevalence of myalgia.

**
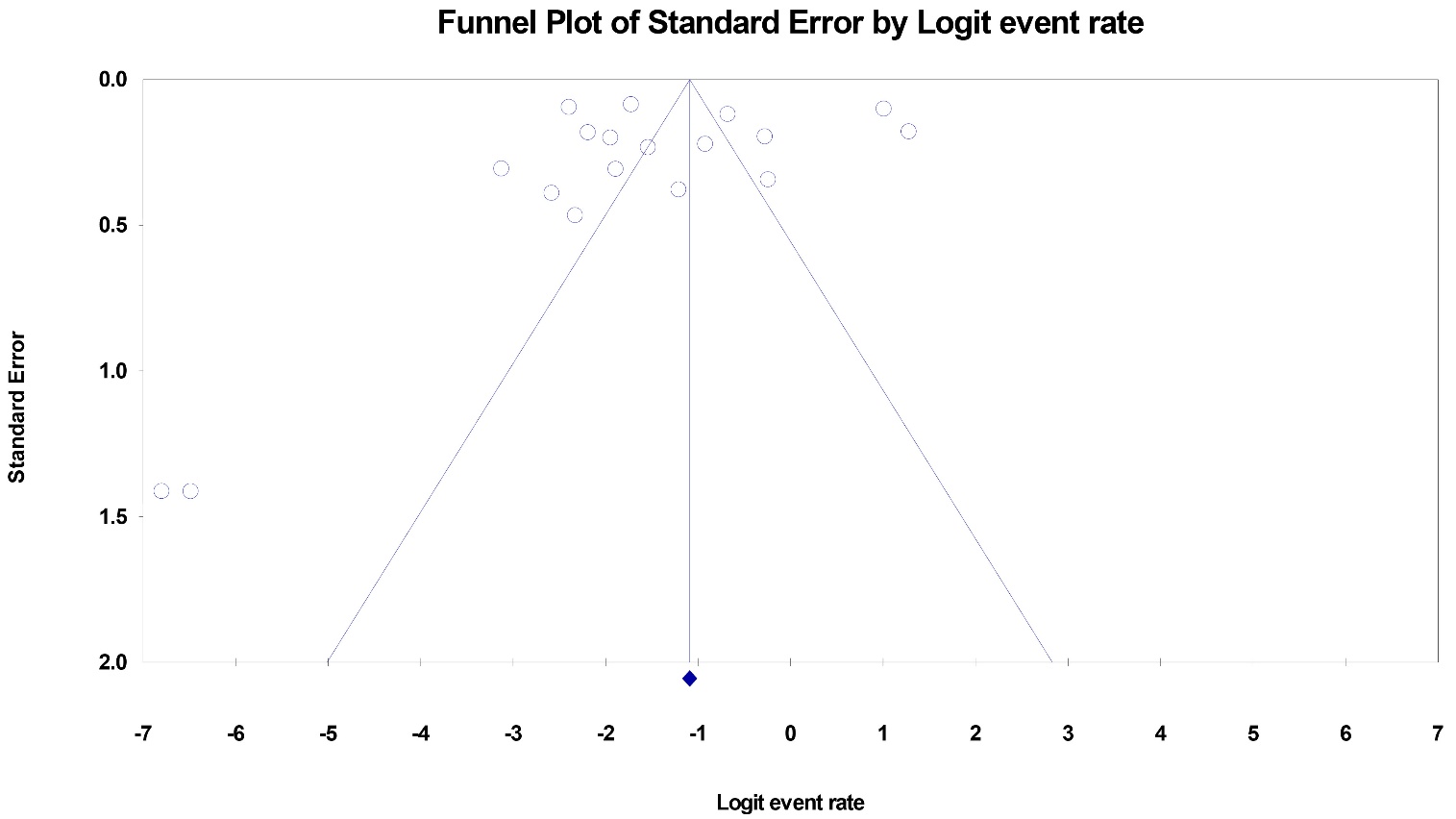
**

Supplementary Fig. 10. Funnel plot for publication bias of the global prevalence of arthralgia.

**
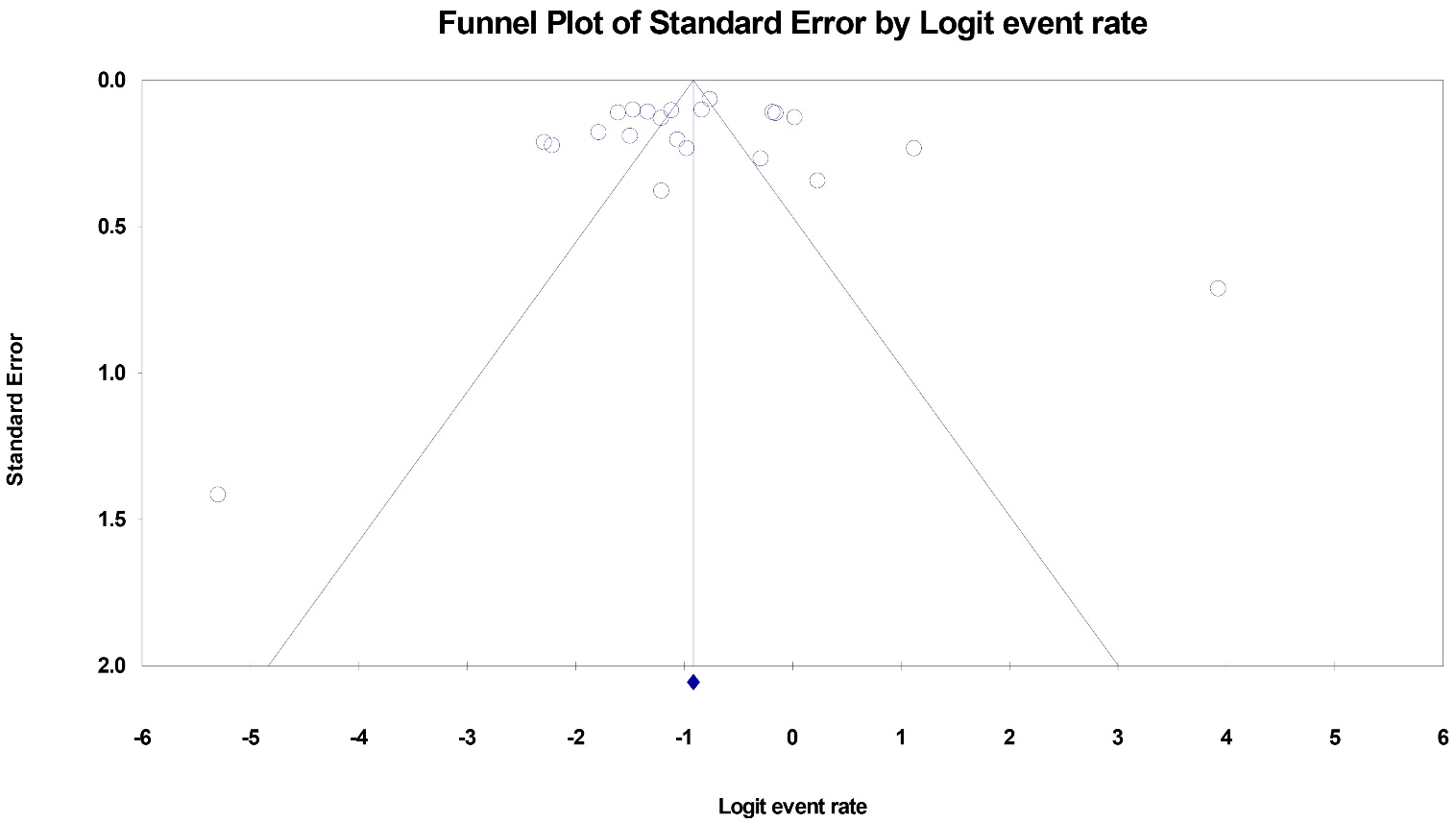
**

Supplementary Fig. 11. Funnel plot for publication bias of the global prevalence of TMJ clicking/sound.

**
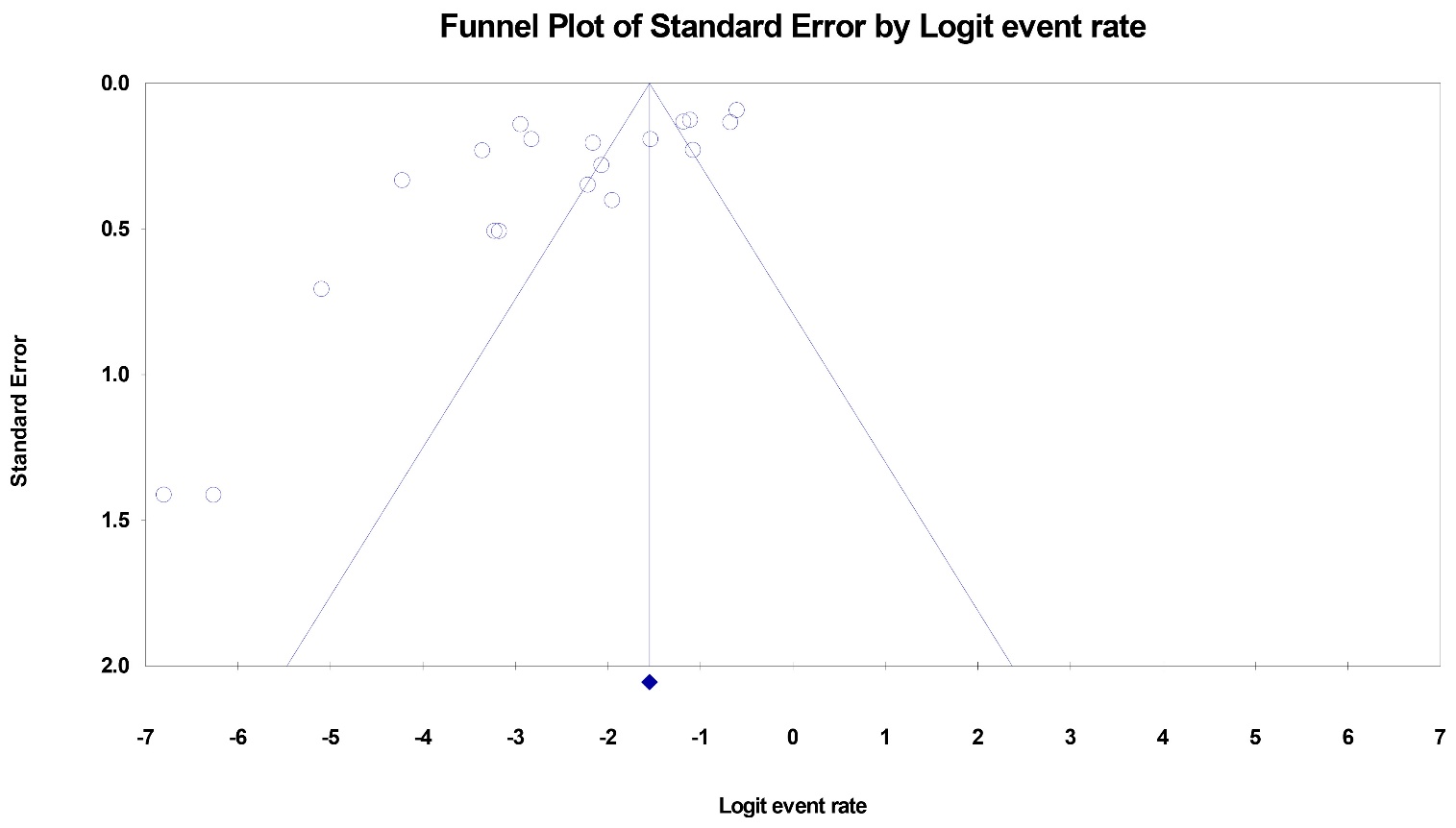
**

Supplementary Fig. 12. Funnel plot for publication bias of the global prevalence of limited mouth opening.
